# Supplementary material for: All-at-once RNA folding with 3D motif prediction framed by evolutionary information
Source: Res Sq. 2025 Mar 26:rs.3.rs-5664139. Preprint. [Version 1] doi: 10.21203/rs.3.rs-5664139/v1 (PMC11974997; doi:10.21203/rs.3.rs-5664139/v1)
Supplement: 1 [file NIHPPrs5664139V1-supplement-1.pdf]

Methods

The RNA Basic Grammar including 3-way and 4-way junctions (RBGJ3J4)

The RNA basic grammar (RBG) [62] is a probabilistic stochastic context-free grammars (SCFG) modeling RNA folding, targeting secondary structural elements including stacked canonical base pairs forming helices, hairpin loops, bulge and internal loops, and multiloops.

SCFGs build correlated states at arbitrary distances and hence are well suited for expressing RNA base pairing. Moreover, the nonterminals directly correspond to the overarching secondary structures. For example, the non-terminal `F` builds a helix, while the non-terminal `P` is present after a helix has ended and is able to initiate all possible loop features such as a hairpin loop, internal loop, or multiloops (Figure S1).

Because many RNA 3D motifs occur in multiloops, especially the most frequent 3- and 4-way junctions, here we introduce the RBGJ3J4 grammar which singles out 3-way junctions (multiloops framed by 3 helices) as well as 4-way junctions (multiloops framed by four helices) as specific cases from any other higher order multiloop. See Figure 2a and Figure S1 for the full description of the RBGJ3J4 grammar.

Another unique feature of the RBG and RBGJ3J4 grammars as they are used in CaCoFold is that they fold an alignment, not a single sequence. A position does not represent one residue but a probability vector describing the frequency of each residue in the aligned column. This way, we can produce consensus secondary structures that include the information contained in all aligned sequences.

The RBGJ3J4-R3D joint grammar

The R3D motif SCFGs are integrated into the RBGJ3J4-R3D grammar as described in Figure 2. CaCoFold-R3D admits an arbitrary number of 3D motifs. Figure S3 describes the list of 51 motifs used in this manuscript. CaCoFold-R3D internally interprets the descriptor lists and implements a SCFG for each motif according to the general R3D grammars for each of the six types of motifs as described in Figure 3.

RNA 3D motifs bound by two or more helices, can appear in different configurations depending on which of the ends corresponds to the $5'/3'$ ends of the molecule, versus all the others for which the backbone is continuous. That is, `BLs` and `ILs` can have two configurations, while `J3s` and `J4s` can have three and four respectively (Figure S2). For a given representation of the motif, R3D internally implements and adds all possible configurations which get added to the RGBJ3J4-R3D grammar.

As with the RBGJ3J4 grammar, all R3D SCFGs describe a consensus motif in an alignment, not just a particular sequence motif. Thus, R3D is able to represent the variability observed in the motif.

CaCoFold-R3D implements the CYK algorithm [65, 10] to report the consensus fold and consensus 3D motifs that maximize the probability of the alignment. The input to the algorithm is not one nucleotide per sequence position, but L probability distributions of dimension 4 describing the frequency of each nucleotide per alignment position (a probabilistic sequence), and $L \times L$ distributions describing join 4×4 pair probabilities.

The sequence-motif profile HMMs

Each sequence motif is characterized by a consensus sequence $S_1 \dots S_L$, and it gets assigned a profile HMM (Figure 3g). The profile HMM introduces one consensus state per position S_i, for $1 \leq i \leq L$. Each consensus state is characterized by a consensus residue or residue type such as: A, or R (A or G), or Y (C or U) or others that determine the emission probability of the consensus. There is an error probability to allow any of the other residues not designated by the consensus. That is a $S = R$ position assigns $P_S(A) = P_S(G) = 0.5 - \epsilon/2$ and $P_S(C) = P_S(U) = \epsilon/2$. The profile HMM also includes one insertion states per position. All inserted position use the same emission residue probability distribution matching the residue frequencies of the training set. Emissions are done on transition, and transitions without emissions describe deletion events.

Each profile HMM is parameterized by a length distribution with an expected length closely matching that of the consensus motif, and allowing insertions and deletions relative to consensus. Empty sequence motifs are also modeled by a profile HMM to allow for the possibility of more divergent motif examples with insertions.

The profile HMMs described in Figure 3g can be used to score either individual sequence positions or alignment positions. Introducing a probability distribution per position o, $\{p_o(a)\}_{a=A,C,G,U/T}$, the consensus and indel emission probabilities of the profile HMM can be written as

$$P_{S_i}(o) = \sum_{a=A,C,G,U} p_o(a) P_{S_i}(a),$$
$$P_{\text{indel}}(o) = \sum_{a=A,C,G,U} p_o(a) P_{\text{indel}}(a).$$

For the case of an aligned position, p_o is the position base composition. For the case of a particular sequence, there is a single residue per position $o = a$, such that $p_o(o = a) = 1$, and

$$P_{S_i}(o = a) = P_{S_i}(a),$$
$$P_{\text{indel}}(o = a) = P_{\text{indel}}(a).$$

For each tested segment (in alignment or sequence), the profile HMM calculates the probability of the segment given the motif using the forward algorithm. The probability for a given subsequence is incorporated into the corresponding R3D SCFG where the sequence segment is included.

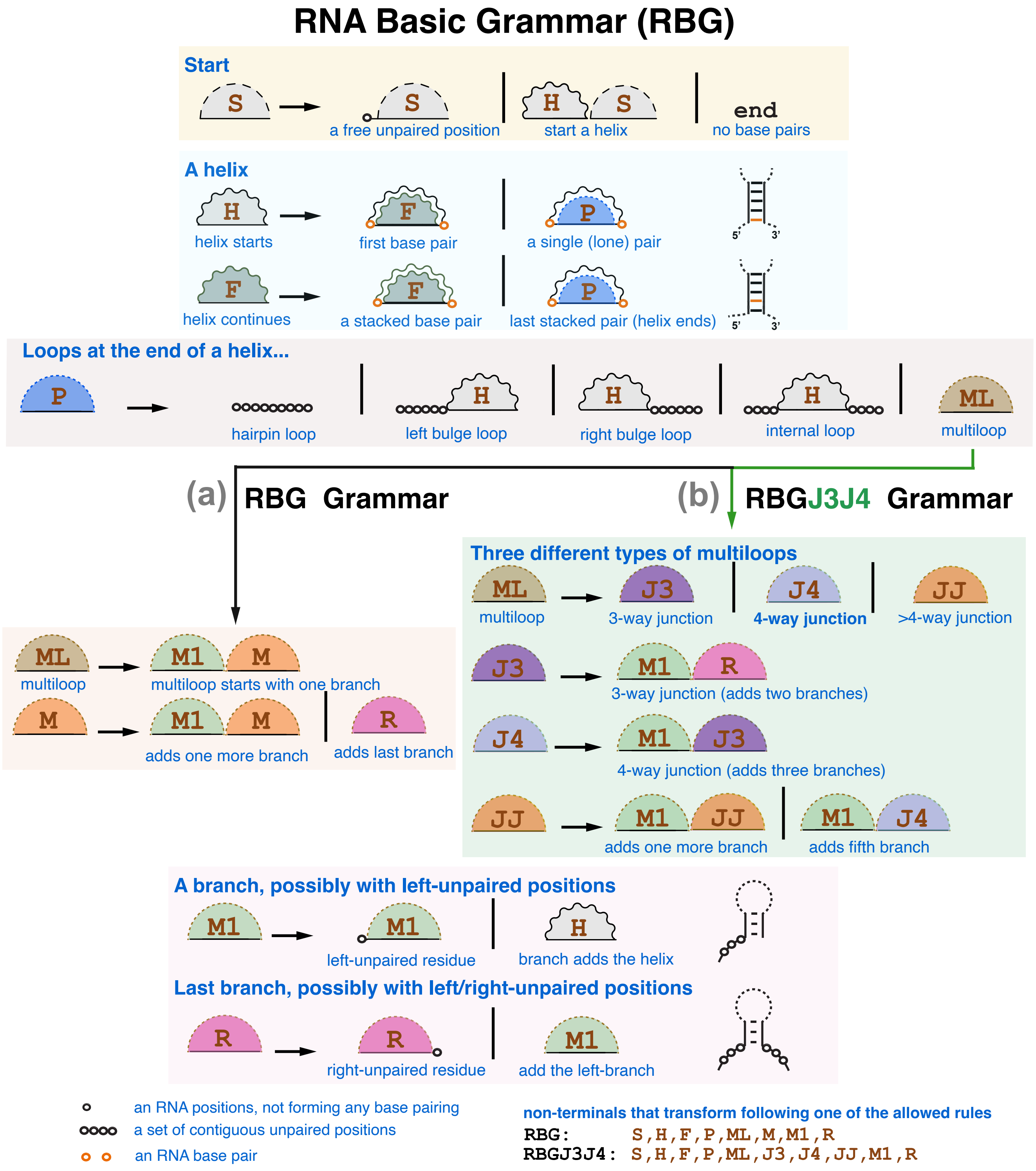

Figure S1: **The RBG and RBGJ3J4 generative grammars.** Inserts **(a)** and **(b)** describe the two distinct realizations of the multiloop non-terminal ML. RBGJ3J4 replaces the generic multiloop non-terminal M with non-terminals J3, J4 and JJ in order to distinguish 3-way and 4-way junctions from other higher order multiloops. A solid line represent the RNA sequence, a curly line indicates that the two connecting residues are base paired, and a dashed line indicates that the relationship between the two residues is yet undetermined. Non-terminals are depicted in brown and actual residues/positions are depicted with circles (black for unpaired and orange for base paired positions). Each non-terminal describes a discrete random variable of events which are enumerated on the right-hand side of the arrows. The allowed events for a given non-terminal are separated by the | ("or") symbol. Starting from the **S** non-terminal, an RNA sequence/structure is produced by sampling from the discrete probability distributions (transitions and emissions) associated to each non-terminal.

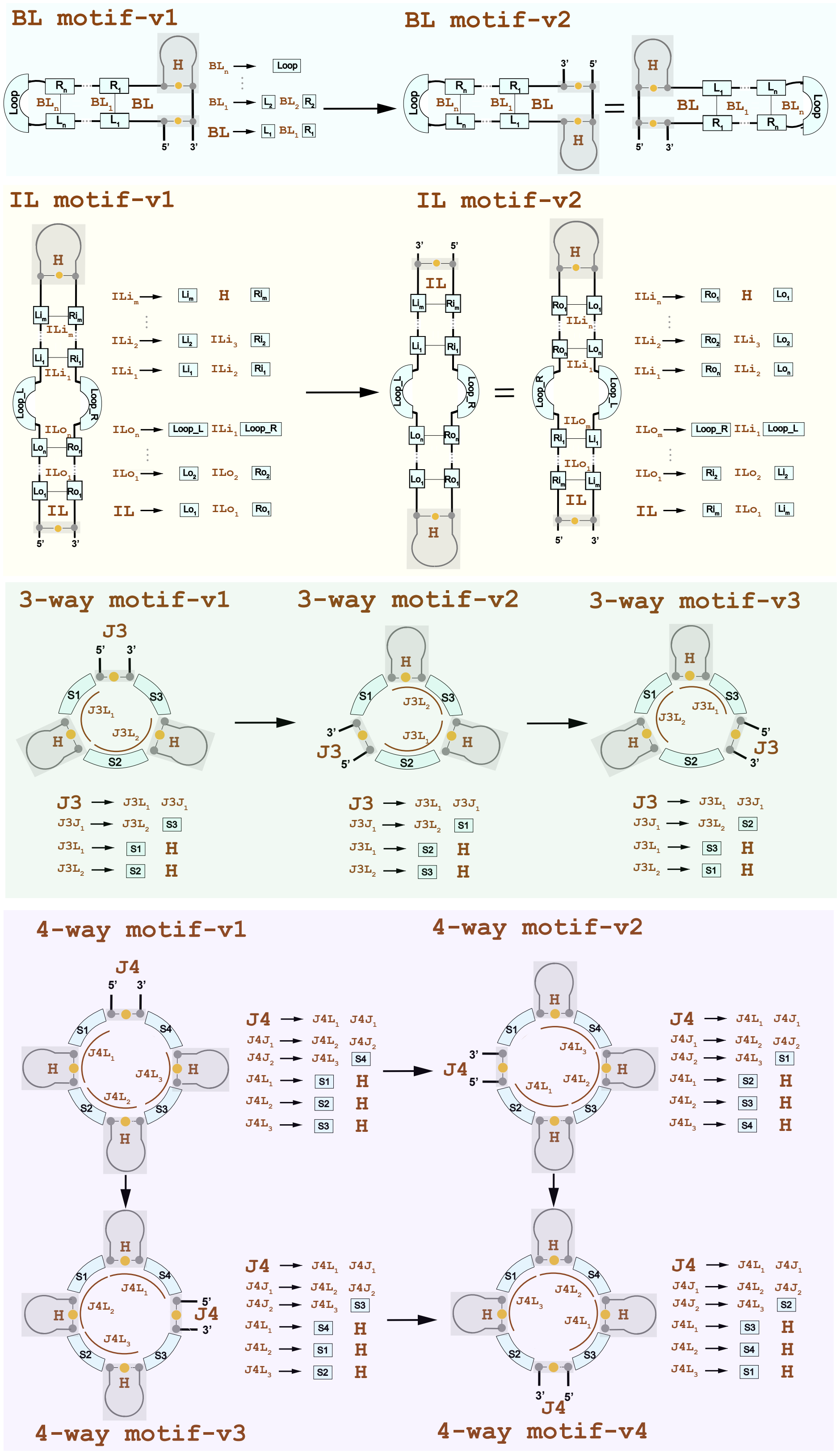

Figure S2: **RNA 3D motifs variants.** RNA 3D motifs bound by more than one helix (*i.e.* all except for hairpin loops) allow different topological variants depending on which $5'/3'$ ends are selected to integrate the motif into the rest of the structure. Bulge and Internal loop motifs have two variants, and 3-way and 4-way junctions have three and four variants respectively. For any 3D motif entry, CaCoFold-R3D internally models all possible variants of the motif.

```
#HL   Loop(5'-3')   L(5'-3')       R(5'-3')      name
#----------------------------------------------------------------------------------------------------------------
 HL   N             G              RA            GNRA-tetraloop
 HL   URA           -              -             U-turn
 HL   UNCG          -              -             UNCG-tetraloop
 HL   ANYA          -              -             ANYA-tetraloop
 HL   CUYG          -              -             CUYG-tetraloop
 HL   YGNN          -              -             YGNN-tetraloop
 HL   GANC          -              -             GANC-tetraloop
 HL   UNAC          -              -             UNAC-tetraloop
 HL   URRR          -              -             T-loop-tetraloop
 HL   UAACR         -              -             L8_RNaseP_bact_a
 HL   AG            UAGUACG        AGGACC        Sarcin-ricin_loop
 HL   AGGAY         -              -             CsrA_binding
 HL   GAGUA         -              -             GAGUA_pentaloop
 HL   AA            -              -             AA-5SrRNA
 HL   GCRYA         -              -             U6-loop

#BL   Loop(5'-3')   L(5'-3')       R(5'-3')       name
#----------------------------------------------------------------------------------------------------------------
 BL   UGRAA         -              -              Docking-elbow
 BL   AA            -              -              AA-bulge
 BL   G             -              -              bulged-G

#IL   Loop-L(5'-3') Loop-R(5'-3')  L-o(5'-3')     R-o(5'-3')     L-i(5'-3')   R-i(5'-3')    name
#----------------------------------------------------------------------------------------------------------------
 IL   -             RNN            N,G,A          N,A,R          -            -             K-turn
 IL   -             RNN            G,A            A,R            -            -             K-turn-b
 IL   G             N              G,A            A,U            U,A          A,G           Loop-E
 IL   UAAG          UAU            C,C            G,G            -            -             GAAA_Tetraloop-receptor
 IL   CVC           V              -              -              -            -             C-loop
 IL   G             -              G,A            A,A            U,A          A,G           G-bulge
 IL   G             -              U,A            C,C            U,A          A,G           G-bulge_Das
 IL   -             -              G,A            A,G            -            -             Tandem-GA
 IL   YCC           AAC            -              -              -            -             Twist-up
 IL   YAA           RAN            -              -              -            -             UAA_GAN
 IL   RR            YN             R              R              A            Y             J4a/4b
 IL   AA            AA             -              -              -            -             J4/5-IL
 IL   GUA           GG             -              -              -            -             GUA^GG_RRE
 IL   CAGG          AGCA           -              -              -            -             S_domain
 IL   AN            -              G,A            A,U            -            -             Hook-turn
 IL   UGRAA         -              -              -              -            -             Docking-elbow-IL
 IL   UU            AUU            -              -              -            -             pK-turn

#J3   S1(5'-3')     S2(5'-3')      S3(5'-3')      name
#----------------------------------------------------------------------------------------------------------------
J3    N             CUGA           A              J3_hammerhead
J3    U             YUCUAC         AC             J3_purine
J3    NN            NNNNNN         NN             J3_typeA
J3    NNNN          NNNN           NNNN           J3_typeB
J3    NN            NNN            NNNNNN         J3_typeC
J3    N             UGAGA          N              J3_TPP
J3    A             RAA            -              J3_groupII

#J4   S1(5'-3')     S2(5'-3')      S3(5'-3')      S4(5'-3')     name
#----------------------------------------------------------------------------------------------------------------
J4    -             AA             -              U             J4_HCV_IRES
J4    N             -              NNN            -             J4_tRNA
J4    N             N              NN             -             J4_manA
J4    R             -              -              R             J4_U1RNA

#BS  Loop(5'-3')      name
#----------------------------------------------------------------------------------------------------------------
BS    UKNRW            T-loop
BS    RRGU             LoopE-a
BS    RARR             LoopE-b
BS    AAYAARAACAANARR  CRC_binding
BS    AGGAY            CsrA_motif
```

Figure S3: **RNA 3D motifs descriptors.** The descriptor file includes 51 different distinct motifs. CaCoFold internally constructs SCFGS for a total of 96 motif variants. The 96 motif R3D SCFGs get integrated into the RBGJ3J4 grammar. The RBGJ3J4-R3D grammar folds and detect the motifs of the RNA simultaneously. HL = Hairpin Loop, BL = Bulge Loop, IL=Internal Loop, J3 = 3-way Junction, J4 = 4-way Junction, BS = Branch Segment.

RFAM SEED ALIGNMENTS
4,178 Families

Type	Motif	# Motif instances with cov support (all)	# Rfam families with motif with cov support (all)	SSU eukarya with support (all)	LSU eukarya with support (all)
HL	GNRA_tetraloop	170 (214)	101 (132)	4 (4)	6 (7)
IL	K_turn	68 (83)	54 (68)	2 (2)	2 (2)
BL	Docking_elbow	59 (94)	55 (83)	2 (2)	2 (5)
J3	J3_groupII	52 (57)	40 (43)	1 (1)	1 (1)
HL	UNCG_tetraloop	48 (78)	42 (69)	1 (1)	0 (0)
J3	J3_typeA	46 (49)	36 (39)	4 (4)	4 (4)
BL	AA_bulge	45 (101)	34 (81)	1 (1)	2 (2)
IL	pK_turn	45 (82)	41 (75)	0 (0)	0 (0)
J3	J3_typeC	43 (45)	42 (43)	0 (0)	1 (1)
J4	J4_U1RNA	40 (44)	29 (31)	0 (0)	3 (4)
IL	UAA_GAN	40 (50)	23 (33)	0 (0)	5 (5)
IL	G_bulge	37 (52)	30 (39)	0 (0)	1 (1)
IL	K_turn_b	35 (50)	32 (47)	0 (1)	1 (1)
J4	J4_HCV_IRES	33 (36)	25 (28)	3 (3)	1 (1)
IL	Hook_turn	32 (49)	28 (45)	0 (0)	3 (3)
IL	Tandem_GA	29 (37)	19 (25)	2 (3)	3 (3)
BS	LoopE_a	28 (33)	15 (20)	1 (1)	4 (4)
BS	T_loop	27 (33)	25 (30)	1 (1)	2 (2)
J4	J4_manA	26 (27)	24 (25)	0 (0)	2 (2)
IL	C_loop	26 (42)	25 (40)	1 (1)	0 (0)
BS	CsrA_motif.rev	26 (30)	23 (27)	2 (2)	1 (1)
HL	UNAC_tetraloop	26 (48)	26 (47)	0 (0)	1 (1)
BS	CRC_binding	26 (34)	18 (22)	5 (5)	0 (0)
J4	J4_tRNA	25 (26)	19 (20)	3 (3)	3 (3)
HL	T_loop_tetraloop	24 (43)	20 (34)	1 (3)	0 (0)
BS	LoopE_b.rev	24 (25)	18 (18)	1 (1)	1 (1)
BS	LoopE_b	21 (22)	15 (16)	1 (1)	2 (2)
BL	bulged_G	20 (43)	18 (37)	0 (0)	0 (0)
BS	T_loop.rev	20 (26)	17 (22)	1 (1)	3 (3)
HL	CUYG_tetraloop	20 (44)	20 (43)	0 (0)	0 (0)
HL	AA_5SrRNA	19 (19)	18 (18)	0 (0)	0 (0)
HL	ANYA_tetraloop	18 (39)	16 (35)	0 (0)	1 (1)
IL	J4a/4b	16 (37)	16 (35)	0 (0)	1 (1)
J3	J3_typeB	16 (16)	14 (14)	2 (2)	0 (0)
J3	J3_hammerhead	16 (27)	16 (26)	0 (0)	1 (1)
HL	L8_RNaseP_bact_a	16 (29)	14 (26)	0 (0)	0 (1)
BS	LoopE_a.rev	16 (21)	13 (17)	0 (0)	1 (1)
HL	U_turn	15 (24)	14 (23)	0 (0)	0 (1)
HL	GANC_tetraloop	15 (28)	14 (26)	0 (0)	2 (2)
BS	CRC_binding.rev	15 (18)	8 (11)	6 (6)	0 (0)
BS	CsrA_motif	15 (18)	13 (16)	0 (0)	0 (1)
J3	J3_TPP	14 (19)	12 (17)	0 (0)	0 (1)
HL	YGNN_tetraloop	13 (27)	12 (26)	0 (0)	0 (0)
IL	GUA_GG_RRE	10 (13)	10 (12)	0 (0)	0 (0)
HL	CsrA_binding	10 (29)	7 (14)	0 (1)	0 (0)
HL	GAGUA_pentaloop	10 (22)	9 (21)	0 (0)	0 (0)
IL	S_domain	10 (21)	10 (20)	0 (0)	0 (0)
IL	Loop_E	9 (11)	9 (10)	0 (0)	1 (2)
IL	Twisted_up	9 (31)	9 (30)	0 (0)	0 (0)
J3	J3_purine	7 (11)	7 (11)	0 (0)	0 (0)
IL	GAAA_Tetraloop_receptor	7 (17)	7 (17)	0 (0)	0 (0)
HL	U6_loop	7 (18)	7 (18)	0 (0)	0 (1)
IL	J4/5_IL	6 (13)	6 (13)	0 (1)	0 (0)
HL	Sarcin_ricin_loop	5 (7)	5 (7)	0 (0)	1 (1)
IL	G_bulge_Das	5 (11)	5 (11)	0 (0)	0 (1)
IL	Docking_elbow_IL	0 (1)	0 (1)	0 (0)	0 (0)

Rfam SEEDS
4,178 alignments

	# Motif instances with cov support (all)	# Rfam families with motif with cov support (all)	SSU eukarya with support (all)	LSU eukarya with support (all)
**3D Motifs all**	1460 (2124)	591 (822)	45 (51)	62 (74)
**Nested helices**	3877 (15395)	1721 (4168)	66 (89)	102 (152)
**PK + higher order**	504 (532)	246 (246)	1 (2)	2 (4)

Rfam CONTROLS
4,178 shuffled alignments

**3D Motifs all**	121 (290)	106 (208)	0 (0)	0 (1)
**Nested helices**	733 (14146)	676 (4149)	0 (34)	0 (75)
**PK + higher order**	175 (188)	102 (102)	0 (0)	0 (0)

Table S1. **RNA 3D motifs found in Rfam.** Results of running CaCoFold-R3D for all seed alignments in the database of structured RNAs Rfam. Results show RNA 3D motif occurrences with covariation support where at least one of the closing helices has at least one covarying base pair, and in parenthesis the total set of predictions. For the other structural elements (nested helices, pseudoknots and other higher order base pair interactions), having covariation support means that the element includes at least one significantly covarying pair of residues. In parenthesis we show the total number of predictions. The control shuffled alignments were obtained by randomizing the residues within each alignment column independently from each other. In these control alignments, covariation between columns is scrambled, but the base composition per column (thus the possible 3D motif identity) remains mostly intact.
